# Formin proteins in megakaryocytes and platelets: regulation of actin and microtubule dynamics

**DOI:** 10.1080/09537104.2018.1481937

**Published:** 2018-06-18

**Authors:** Malou Zuidscherwoude, Hannah L.H. Green, Steven G. Thomas

**Affiliations:** 1 Institute of Cardiovascular Sciences, University of Birmingham, Birmingham, UK; 2 Centre of Membrane Proteins and Receptors (COMPARE), University of Birmingham and University of Nottingham, Midlands, UK

**Keywords:** Actin, Formin, macrothrombocytopenia, microtubules cytoskeleton, proplatelet formation

## Abstract

The platelet and megakaryocyte cytoskeletons are essential for formation and function of these cells. A dynamic, properly organised tubulin and actin cytoskeleton is critical for the development of the megakaryocyte and the extension of proplatelets. Tubulin in particular plays a pivotal role in the extension of these proplatelets and the release of platelets from them. Tubulin is further required for the maintenance of platelet size, and actin is the driving force for shape change, spreading and platelet contraction during platelet activation. Whilst several key proteins which regulate these cytoskeletons have been described in detail, the formin family of proteins has received less attention. Formins are intriguing as, although they were initially believed to simply be a nucleator of actin polymerisation, increasing evidence shows they are important regulators of the crosstalk between the actin and microtubule cytoskeletons. In this review, we will introduce the formin proteins and consider the recent evidence that they play an important role in platelets and megakaryocytes in mediating both the actin and tubulin cytoskeletons.

## Formins

### Actin filament nucleation and elongation

**Figure 1.**
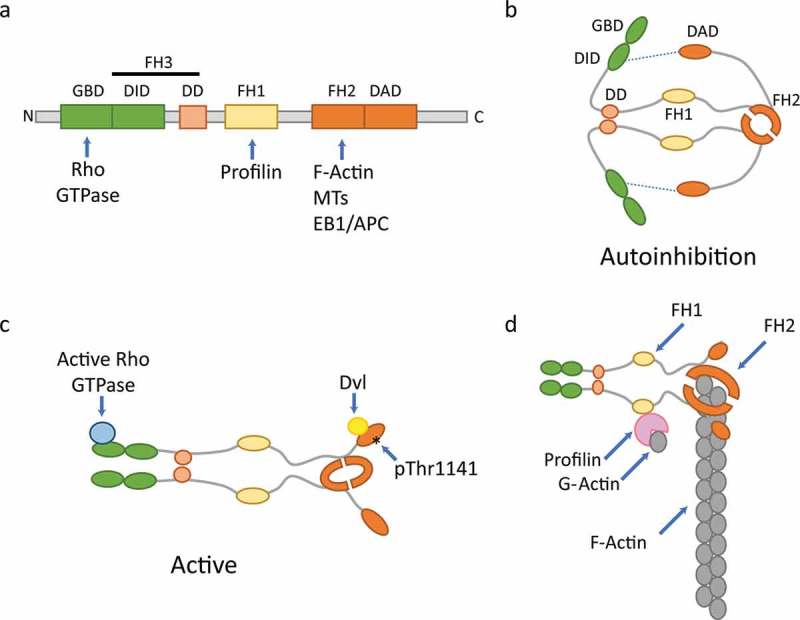
Generalised schematic of formin domains and structures. **a)** Typical domains present in members of the formin family and binding sites for proteins which interact with formins. GBD = GTPase binding domain, DID = diaphanous inhibitory domain, DD = dimerisation domain, FH1 = formin homology 1, FH2 = formin homology 2, FH3 = formin homology 3, DAD = diaphanous auto-regulatory domain. **b)** The homodimer is formed through interactions of the DD and FH2 domains on each half of the dimer with auto-inhibition being achieved by interaction of the DAD with DID domains (blue dashed line). **c)** The active conformation is achieved following binding of GTP-bound Rho GTPases to the GBD, via binding to dishevelled (Dvl) (DAAM1) or Rho kinase mediated phosphorylation at Thr^1141^ (FHOD1). **d)** Formins nucleate linear actin filaments by binding of dimerised FH2 domains to actin dimers. Elongation of the filament occurs via processive capping; the binding of profilin:ATP G-actin to the FH1 domain allows the formin to facilitate addition of actin monomers to the growing actin filament whilst protecting it from capping proteins and depolymerisation. Figure adapted from (11,68).

Formin proteins were first identified in mice from studies on abnormal limb development (1,2) with homologues subsequently being found in drosophila and yeast (3). A list of the formin proteins expressed in mammalian cells is given in Table I. Formins have the ability to both nucleate and accelerate the elongation of linear actin filaments. They play a crucial role in the assembly of cytoskeletal structures such as filopodia, lamellipodia and stress fibres and are therefore required for a range of cellular processes including cell adhesion, cell division and cell motility (4). These multidomain proteins are defined by the presence of highly conserved Formin Homology 1 (FH1) and Formin Homology 2 (FH2) domains and a less well-conserved FH3 domain (Figure 1(a)). Formins function as a homodimer with dimerisation mediated via the dimerisation domain (DD), a component of the FH3 domain (5) and the FH2 domains (Figure 1(b)). The FH2 domains form a doughnut-shaped head-to-tail dimer which nucleates actin filaments. Indeed, it has been shown that the FH2 domains alone are sufficient for actin filament nucleation probably via the stabilisation of spontaneously formed actin dimers (6). The FH2 domain remains processively attached to actin filament barbed ends facilitating addition of actin monomers to support elongation and shielding the filament from abundant capping proteins (7–9). The FH1 domains recruit profilin bound G-actin molecules, bringing them into close proximity with the barbed end and thus accelerating filament elongation (10) (Figure 1(c)).

**Table I.** Mammalian formin proteins and their abundance in human and mouse platelets.

Full protein name	Abb. protein name^1^	Human Uniprot ID^2^	Mouse Uniprot ID^2^	Copies per human platelet^3^	Copies per mouse platelet^4^	Human platelet transcript abundance^5, 6^	Mouse platelet transcript abundance^5, 6^
Dishevelled-associated activator of morphogenesis 1	DAAM1	Q9Y4D1	Q8BPM0	3800	8,529	3.53	6.47
Dishevelled-associated activator of morphogenesis 2	DAAM2	Q86T65	Q80U19	ND	ND	0.04	0.00
Protein diaphanous homolog 1	DIAP1 (mDia1)	O60610	O08808	8100	14,408	92.77	55.13
Protein diaphanous homolog 2	DIAP2 (mDia2)	O60879	O70566	ND	ND	0.11	0.02
Protein diaphanous homolog 3	DIAP3 (mDia3)	Q9NSV4	Q9Z207	ND	ND	0.04	0.06
FH2 domain-containing protein 1	FHDC1	Q9C0D6	Q3ULZ2	ND	ND	0.02	0.13
FH1/FH2 domain-containing protein 1	FHOD1	Q9Y613	Q6P9Q4	7500	21,598	7.84	2.71
FH1/FH2 domain-containing protein 3	FHOD3	Q2V2M9	Q76LL6	ND	ND	0.00	0.00
Formin-1	FMN1	Q68DA7	Q05860	ND	ND	0.03	0.00
Formin-2	FMN2	Q9NZ56	Q9JL04	ND	ND	0.00	0.00
Formin-like protein 1	FMNL1	O95466	Q9JL26	ND	ND	4.73	0.02
Formin-like protein 2	FMNL2	Q96PY5	A2APV2	ND	ND	0.09	0.00
Formin-like protein 3	FMNL3	Q8IVF7	Q6ZPF4	ND	34	0.91	2.41
Delphilin	Delphilin	A4D2P6	Q0QWG9	ND	ND	0.00	0.00
Inverted formin-2	INF2	Q27J81	E9QLA5	6600	14,269	99.89	162.86

^1^Abbreviated protein names taken from Uniprot and used in this review. Name in brackets is the commonly used name which we used in this review

^2^http://www.uniprot.org/

^3^Data from Burkhart *et al*. (34)

^4^Data from Zeiler *et al*. (38)

^5^Data from Rowley *et al*. (37)

^6^Mean RPKM (Reads Per Kilobase of transcript per Million mapped reads)

### Activation of formin proteins

Formin proteins exist in an auto-inhibited state. Diaphanous-related formins (DRFs) contain conserved regulatory domains; a C-terminal Diaphanous Auto-regulatory Domain (DAD) and an N-terminal Diaphanous Inhibitory Domain (DID – which forms part of the FH3 domain) (11). Intramolecular interactions between the DAD and DID lead to auto-inhibition of formin activity (Figure 1(d)). As a general rule, this auto-inhibition can be relieved upon the interaction of the GTPase-binding domain (GBD) with an active Rho GTPase (12). Therefore, Rho GTPases recruit formins to different locations within the cell for localised actin filament assembly.

DIAP1 (also known as DIAPH1, but commonly known as mDia1) is the most studied formin and was found as an effector of the small GTPase RhoA in the formation of actin stress fibres (13). In its active form, it is a potent actin nucleator and accelerates filament elongation. mDia1 is activated as described above by GTP bound Rho GTPase binding to the GBD causing displacement of the DAD domain from the N terminal region of mDia1. However, other members of the formin protein family show different mechanisms of DAD release; for example, DAAM1 is activated when the PDZ domain of the Dishevelled (Dvl) protein binds to the DAD domain rather than by Rho binding (14). Similarly, although FHOD1 is recruited to the plasma membrane by active Rac1 binding, this formin seems not to be activated by GTPase interactions. Instead the auto-inhibitory state is disturbed by the phosphorylation of residues in its DAD domain by Rho effector kinase ROCK (15) and cGMP-dependent protein kinase 1 (PRKG1) (16).

Differences also exist in the activity and functions of individual formins in cells. In comparison with mDia1, FHOD1 is less effective at processively elongating actin filaments, but rather acts as an actin bundling factor while protecting filaments from depolymerisation (17,18). INF2 has a Wiskott–Aldrich syndrome homology region 2 (WH2) domain which replaces the DAD found in the DRFs and is able to bind monomeric actin. Interestingly, INF2 is able to sever filaments and accelerate actin filament depolymerisation on the pointed end by virtue of its WH2 domain, as well as to nucleate and elongate actin via its FH2 domain. The switch between polymerisation and depolymerisation activity is thought to be mediated by the hydrolysis of bound ATP within actin subunits upon addition to a filament and the subsequent release of phosphate (19,20).

### Microtubule organisation and dynamics

**Figure 2.**
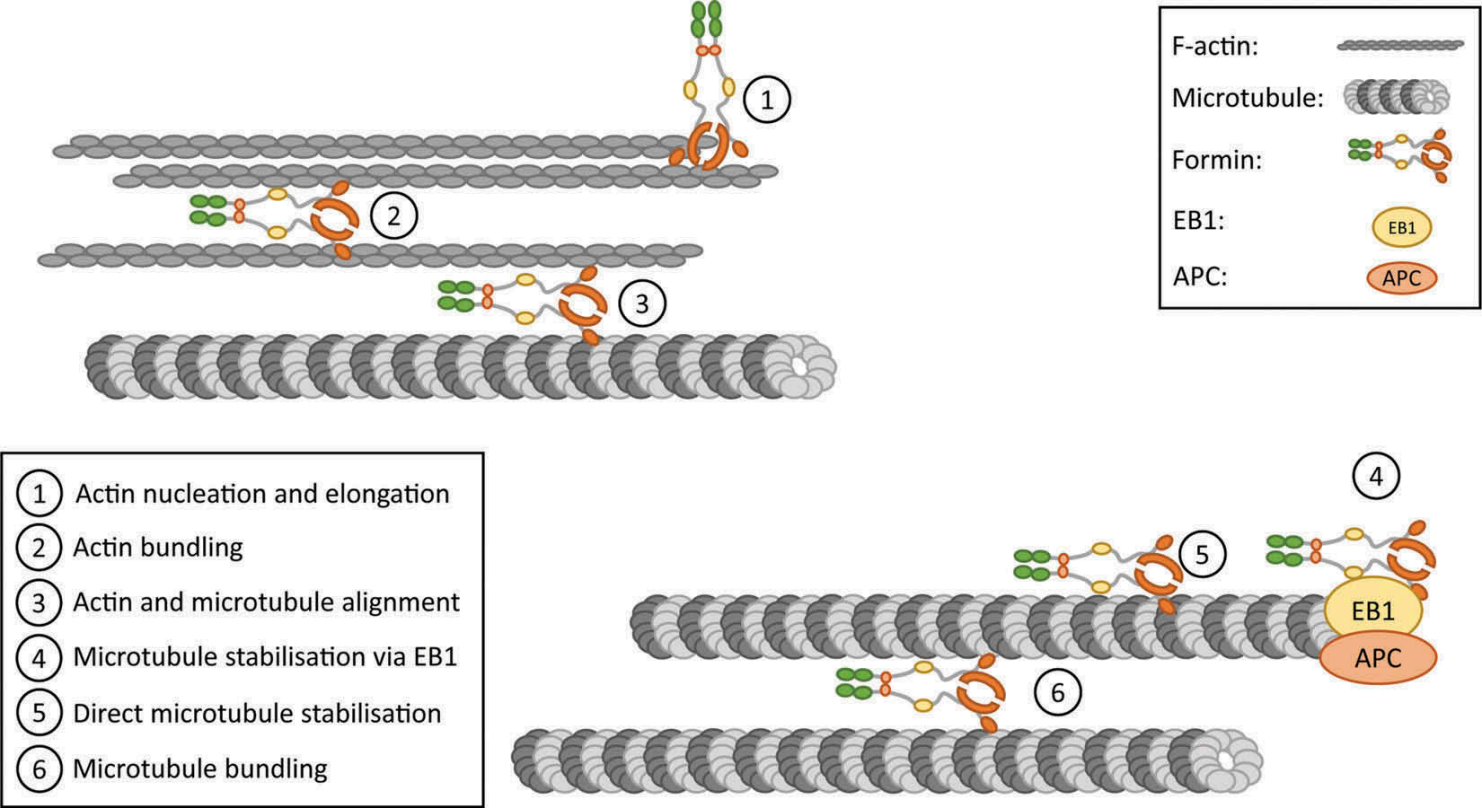
Mechanisms of actin and microtubule regulation and coordination by formins. Formins are proposed to have direct and indirect actions on actin filaments and microtubules. **1**. Actin nucleation and elongation by formins occurs by processive capping of linear actin filaments. **2**. Formins also bundle actin filaments together and **3** align them with microtubules. **4, 5**. Formins can bind to and stabilise microtubules directly or via interaction with capping proteins including EB1. **6**. Like for actin filaments, microtubules can also be brought in close proximity to each other by bundling regulated by formins. Figure adapted from (69,70).

In addition to actin filament assembly, formins regulate microtubule organisation and dynamics, providing a means of crosstalk between the two cytoskeletons. Initial evidence for this came from studies whereby activation of endogenous mDia1 by overexpression of a DAD domain led to a stabilisation of microtubules (21). mDia1 has been shown to interact with microtubules through its FH2 domain. This interaction is via both direct binding to microtubules and through the microtubule plus end tracking proteins EB1, APC 100 and CLIP170 (22). Henty-Ridilla *et al*. found that CLIP170 increased the elongation rate of actin filaments assembled by formins including DAAM1 and INF2 and that a complex of mDia1/CLIP170 and EB1 triggered accelerated actin polymerisation from microtubule plus ends (23). The strength and effects of interactions between FH2 domains and microtubules vary depending upon the source of the FH2 domain. For example, the FH2 domain from mDia2 binds with a 1:1 stoichiometry to tubulin whereas that from INF2 and mDia1 binds with a 1:3 stoichiometry (24). In addition, binding of microtubules to mDia2 FH2 domains strongly inhibits actin polymerisation where binding has no effect of INF2 FH2 domains (24). Furthermore, DAAM1 can bind to both actin and microtubules simultaneously linking actin polymerisation to regions of stable microtubules (25) and co-alignment of actin filaments and microtubules is observed when active fragments of mDia1 and FHOD1 are expressed (26,27). Microtubule stability can also be regulated by post-translational modification of alpha tubulin via acetylation and detyrosination (28,29), and it has been demonstrated that FH1-FH2 domains from the majority of formin proteins can induce the formation of stabilised microtubules through these post-translational modifications (30,31). Finally, although it has been shown that regulation of microtubule stability by formins can be independent of their actin polymerisation activity (30), it is likely that *in vivo* these functions are closely coordinated. Indeed, more recently, it has been suggested that different formins could work in series or in collaboration to regulate microtubule stability or actin assembly as evidenced for mDia1 and INF2 (32). The proposed role of formins in actin and microtubule organisation is summarised in Figure 2.

## Expression of formins in megakaryocytes and platelets

In mammals, the family of formin proteins contains 15 members which are subdivided into eight groups based on their protein domain architecture. The formins vary in their localisation, binding partners and regulation, and show differences in the strength and efficiency of their actin nucleation and elongation activity (11) indicating that they may play many specialised roles in different cell types. Of these 15 formins only a subset are expressed in platelets and megakaryocytes.

**Figure 3.**
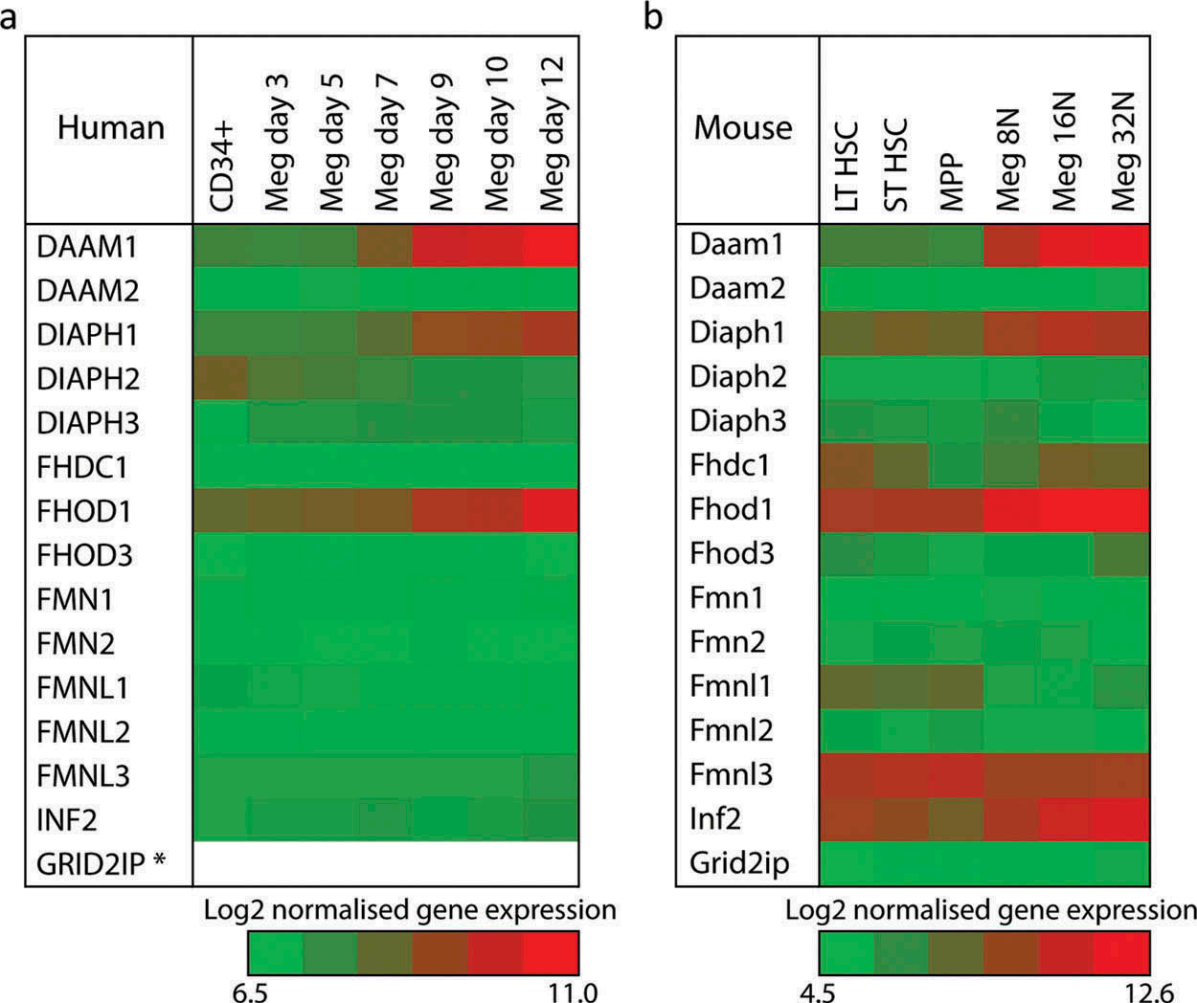
Expression of mammalian formin proteins during megakaryocyte development. **a)** Relative expression levels of the 15 mammalian formin proteins during the development of human megakaryocytes. CD34+ cells from umbilical cord blood were cultured in vitro in the presence of thrombopoietin and interleukin 1β and gene expression quantified by RNA-seq. Data are taken from the Blueprint epigenome project (http://www.blueprint-epigenome.eu/) and (35). * Note – no data was available for the expression of GRID2IP. **b)** Relative expression levels of the 15 mammalian formin proteins during the development of mouse megakaryocytes. Bone marrow cells from C57BL/6 mice were sorted using FACS and gene expression quantified by microarrays. Data are taken from the haemosphere database (http://haemosphere.org/) and (36). For both panels, Log2 of normalised gene expression is represented by a heat map where red equals increased gene expression. Gene names in this figure and throughout the review use the conventions of the HUGO Gene Nomenclature Committee (www.genenames.org) and the Mouse Genome Informatics (www.informatics.jax.org) for human and mouse genes, respectively.

Thomas *et al*. had previously reported on formin expression in platelet and megakaryocytes using SAGE and Haematlas databases (33). Since this time, further studies and advances in technology have allowed for more accurate measurements of the formins at both the gene and protein expression level in platelets and megakaryocytes (34–38). In these genomic and proteomic studies, the expression profile of the mammalian formin proteins changes over the developmental time course of the megakaryocyte. In humans, expression of all formin proteins is low in CD34+ progenitor cells. Over the course of progenitor cell commitment to the megakaryocyte lineage and differentiation into mature cells, the formins DAAM1, DIAPH1 (mDia1) and FHOD1 all significantly increase their expression (Figure 3(a)). A similar pattern is observed in mouse cells with a few differences (Figure 3(b)), i). Fmnl3 appears highly expressed in the LT- and ST-HSC and its expression decreases during differentiation – in mature megakaryocytes, there is still some expression of this formin, ii) there appears to be a small increase in expression of Fhdc1 in maturing mouse megakaryocytes and iii) Inf2 increases in its expression during differentiation.

In platelets, studies have used mass spectrometry-based proteomics and RNA transcript abundance to assess the levels of the formin proteins in human and mouse platelets (Table I). In human platelets, DAAM1, mDia1 and FHOD1 are all found at the protein level, matching closely the gene expression data from megakaryocytes. However, interestingly, INF2 is also found in human platelets despite no expression being reported in megakaryocytes and there is no evidence for mDia2 expression in human platelets even though it has been shown previously by immunoprecipitation and Western blot (33). This protein expression pattern is also reflected in the RNA transcript abundance in the platelets, with the addition of RNA transcripts for Fmnl1 being observed, despite no evidence for expression at the gene of proteins level in studies in developing megakaryocytes and platelets.

In mouse platelets, again DAAM1, mDia1 and FHOD1 are all found to be expressed along with INF2, which in this case matches the megakaryocyte expression data. This pattern is repeated for the platelet RNA transcript levels. A very small amount of Fmnl3 protein and transcript is reported and this is likely to be a carry over as this formin is expressed early in differentiation. Taken all together, data from the genomics, proteomics and other studies indicate that DAAM1, mDia1 and FHOD1 are the major formin proteins in platelets, with a previously unidentified expression of INF2, particularly in mouse platelets. The presence of mDia2 in human platelets is unclear.

## Roles of formin proteins in megakaryocytes and platelets

The formins mDia1 and DAAM1 were first identified in human platelets by RhoA affinity chromatography with the affinity for active RhoA (RhoA-GTP) being approximately 100× (mDia1) and 200× (DAAM1) higher than for inactive RhoA (RhoA-GDP). Both formins were shown to associate with RhoA in platelets activated with thrombin and were both able to initiate actin polymerisation in platelet lysates (39). Subsequently, it was shown that mDia1 moved to the cytoskeleton (Triton insoluble fraction) in activated platelets in a PI3-kinase dependent manner (40). Work by Steele *et al*. studying Wnt3a signalling in platelets has demonstrated that DAAM1 associates with dishevelled (Dvl), an effector of Wnt signalling, upon activation by TRAP and therefore may be important in the activation of RhoA via RhoGef recruitment (41). Thomas *et al*. also identified that FHOD1 was activated in mouse and human platelets downstream of various platelet agonists in a Rho Kinase (ROCK) dependent and Rac1 independent manner (33).

## mDia1 function in platelets and megakaryocytes

Formin protein function has been investigated using knockout mouse models, primarily for mDia1. Two independent labs have created Diaph1 (mDia1) null mice (42,43). Both models are apparently healthy and breed normally but display consistent defects in white blood cells including lymphopenia, myeloproliferative syndrome, myelodysplastic syndrome and reduced adhesion, migration and chemotaxis of neutrophils and lymphocytes (42–47). Studies on the platelets of the mDia1 knockout mice revealed no major phenotype in terms of activation, aggregation or spreading (33). We have tested platelet function following the global inhibition of formin FH2 domains using the small molecule inhibitor SMIFH2 and found that it blocks platelet spreading and cytoskeletal organisation, indicating that formin activity is required for platelet spreading (Thomas, Unpublished data). The reason for the lack of phenotype in mDia1 KO platelets is unclear, but is presumably due to functional redundancy with other formin proteins; in the milieu of the activated platelet, it is highly likely that the abundance of free actin barbed ends, profilin bound G-actin and activated Rho family small GTPases allows for formin mediated actin assembly, even in the absence of one of the formin family members. Alternatively, the lack of phenotype in mDia1 KO platelets could be because mDia1 plays a very specific minor role in the platelet or may primarily play a role in megakaryocytes which effects are carried over in platelets. These functions may not manifest themselves during platelet activation, but affect the resting platelet. Examples of these specific roles include the organisation of actin filaments relative to microtubules in the resting platelet or in regulating the balance of tubulin post-translational modifications that influence microtubule stability and dynamics. This hypothesis is supported by the observation that in our hands, mDia1 knockout mice have an increased platelet size, but normal platelet count (Thomas, Unpublished data) indicating either a defect in platelet formation or an impairment of the ability of the microtubule coil to maintain normal platelet size. This hypothesis, however, remains to be tested.

mDia1 function has also been directly investigated in megakaryocytes. Pan *et al*. (48) used shRNA in human CD34+ derived megakaryocytes to knockdown mDia1 expression to approximately 50–60% of controls. In these cells, proplatelet formation (PPF) was increased, F-actin polymerisation was decreased and the stability of microtubules was increased, as assessed by increased Glu-tubulin (28,29). These data indicate that partial inactivation of mDia1 activity in mature megakaryocytes is required to allow proplatelet formation. This effect is proposed to be mediated through reducing actin-myosin contractile forces in the mature megakaryocyte which allows for the protrusion of proplatelets, an effect also seen in inhibition of myosin IIa (48). However, this effect may also be related to observed changes in microtubule stability which could alter motor protein interaction with microtubules facilitating proplatelet extension (49). Intriguingly, platelet counts are normal in the mDia1 knockout mouse (Thomas, Unpublished data) indicating a possible difference between human and mouse cells, or some compensatory mechanism in the knockout. As indicated above, the mDia1 knockout platelets are increased in size (Thomas, Unpublished data) suggesting that there is a defect in proplatelet formation/release when mDia1 is absent. In addition, the knockdown experiments performed by Pan *et al*. were done after megakaryocyte differentiation, and so, the effect of reduced mDia1 expression on human megakaryocyte development was not studied.

In addition to the loss of mDia1, Pan *et al*. also investigated the expression of constitutively active (CA) mDia1 (just the FH1 and FH2 domains) (48). In these cells, a decrease in PPF formation, an increase in F-actin polymerisation and stress fibre formation was observed (48) which mirrored the results obtained in the mDia1 knockdown cells. More recently, Stritt *et al*. have identified human patients with a gain of function mutation in the auto-regulatory domain of DIAPH1 (R1213*) which leads to a constitutively active protein (50). Patients with the R1213* variant display macrothrombocytopenia and their platelets have abnormal distributions and size of α-granules, vacuoles and membrane complexes (50). Similar to that observed by Pan *et al*., the R1213* variant caused a decrease in PPF in CD34+ derived megakaryocytes providing support to the hypothesis that formin mediated F-actin polymerisation can reduce proplatelet formation due to actin-myosin contractile forces reducing protrusion (as described above). It also indicates that proper regulation of formin activity is required for correct delivery of granules, etc. to the newly forming platelets.

A key difference between the two studies of CA mDia1 is related to their effect on microtubule stability in megakaryocytes and platelets. Pan *et al*. identified that CA mDia1 gave rise to microtubules with decreased stability, whereas Stritt *et al*. observed more stable microtubules. Indeed, the finding of Pan seems to go against the general ability of FH1-FH2 domains to increase microtubule stability (30,31). This difference may well be due to the differences in the constitutively active mDia1 used; the mDiaΔN3 used by Pan *et al*. has additional N terminal deletions compared to the R1213* which may be important in determining how formins interact with microtubules. For example, loss of the Rho-GTPase binding domain in mDiaΔN3 CA mDia1 may potentially cause it to be mis-localised and therefore limit its ability to increase microtubule stabilisation. Likewise, R1213* mDia1 may also lose the ability to be correctly localised within the platelet which would affect its interaction with actin and microtubules. Indeed, in patient platelets, R1213* variant mDia1 displayed altered cellular localisation and gave rise to increased F-actin content and disorganised microtubule organisation (50). Additional families with the R1213* mutation have been identified and a further mutation in the auto-regulatory domain of mDia1 has been reported (A1210Serfs*31), both of which display thrombocytopenia (51) although the actin and microtubule organisation has not been reported in these cases. Finally, although there were clear differences in R1213* platelet cytoskeletons, there was no observed difference in platelet aggregation to a range of agonists (50). This data, taken in light of the mDia1 knockout mouse phenotype, strongly suggest that mDia1 is redundant in platelet activation and its major role is in the formation of platelets from the megakaryocyte.

## Other formin family members in platelets and megakaryocytes

Knockout mice have been generated for other formin proteins (DAAM1, DAAM2, FMN2, mDia2, FHOD3 and INF2) and a range of phenotypes described ranging from cardiac development phenotypes (52–55), infertility (56,57), impaired red blood cell production (58–60) and kidney development and function (61,62). However, despite this, little is known about the effect of loss of these formin proteins on megakaryocyte and platelet function. It is likely that there is significant functional redundancy between the formins or in megakaryocytes and platelets and so phenotypes may be masked by the presence of other formin proteins. Additionally, there may be genetic compensation for loss of protein function which would supress the effect of a harmful mutation (63). A systematic knockout of all four formins expressed in megakaryocytes and platelets would help to answer this but would be an expensive and complicated study. As can be seen from the Stritt study (50), a useful avenue of research is through identifying mutations in formin proteins that give rise to thrombocytopenias and/or platelet function disorders via patient genotyping studies. Many mutations will give rise to a protein with an altered function, rather than complete loss of the protein and so may allow more subtle phenotypes to be identified.

## Future perspectives

As discussed above, the formins are an important family of actin and microtubule regulatory proteins whose ability to i) nucleate and elongate actin filaments, ii) bundle and organise both actin filaments and microtubules and iii) regulate the stability and interactions of microtubules, places them at the centre of cellular cytoskeletal organisation. In relation to platelet function, global inhibition of formins prevents platelet spreading indicating their importance in platelet actin dynamics, however, as of yet, the precise contribution of each of the expressed formins to platelet actin dynamics has yet to be elucidated. In terms of the megakaryocyte, mDia1 has been shown to be important for proper platelet formation, with changes in platelet size, number or morphology being reported for both loss and over-activation of mDia1. Thus, mDia1 likely mediates platelet both the actin and tubulin cytoskeletons during platelet formation where actin-myosin contractility and stable microtubules for proplatelet extension need to be carefully balanced. The roles of Daam1, Fhod1 and INF2 in platelets and megakaryocytes remain to be determined and will require either knockout mouse models, identification of disease causing mutations or induced pluripotent stem cell line studies to understand their functions.

The different classes expressed in megakaryocytes and platelets suggest that they may be playing different roles in the function of these cells. Furthermore, the upstream signalling proteins that activate formins are different depending on the formin (e.g. mDia1 is activated by RhoA, Fhod1 by ROCK and Rac1 and Daam1 by binding to Dvl). These differences indicate that formins may be involved with different processes or in different subcellular localisations in megakaryocytes and platelets which remain to be uncovered. In addition, the downstream effects of formin activation may be specific. For example, mDia1 has been demonstrated to be required for formation of the mother actin filaments upon which ARP2/3 complex can generate lamellipodial protrusions (64) and studies reviewed here have highlighted their key role in coordinating microtubule dynamics. Thus, this may be a mechanism by which mDia1 specifically coordinates the initiation of proplatelet extensions, whereas other formins may regulate different megakaryocyte developmental pathways, for example, regulating the actin pool during endomitosis. In addition, the demonstration that profilin can regulate platelet microtubule dynamics most likely via its interaction with formin proteins (65) highlights the complex and important role that formins may have in regulating actin and microtubules in megakaryocytes and platelets. It is also known that the 3D environment and particularly matrix stiffness is important for regulating megakaryocytes and platelet function (66). Recent observations that mDia1:RhoA can act as a major biochemical switch for mechanosensing/mechanotransduction (67) highlights that in the physiological environment formins may be more important for proper function and as such strides should be taken towards studying them within bone marrow megakaryocytes or within platelets in thrombi. Studies such as these will be important as we consider the causes of uncharacterised thrombocytopenias and bleeding diatheses in patients or as we strive to improve the *in vitro* production of platelets for clinical use, where subtle modifications of proteins that regulate proplatelet formation could increase platelet yields or quality.

## References

[1] Mass RL, Zeller R, Woychik RP, Vogt TF, Leder P. Disruption of formin-encoding transcripts in two mutant limb deformity alleles. Nature. 1990;346:853–855. doi:10.1038/346853a0. 2392151

[2] Woychik RP, Maas RL, Zeller R, Vogt TF, Leder P ‘Formins’: proteins deduced from the alternative transcripts of the limb deformity gene. Nature. 1990;346:850–853. doi:10.1038/346850a0. 2392150

[3] Castrillon DH, Wasserman SA Diaphanous is required for cytokinesis in Drosophila and shares domains of similarity with the products of the limb deformity gene. Development. 1994;120:3367–3377. 7821209 10.1242/dev.120.12.3367

[4] Bartolini F, Gundersen GG Formins and microtubules. Biochim Biophys Acta. 2010;1803:164–173. doi:10.1016/j.bbamcr.2009.07.006. 19631698 PMC2856479

[5] Otomo T, Otomo C, Tomchick DR, Machius M, Rosen MK Structural basis of Rho gtpase-mediated activation of the formin mDia1. Molecular Cell. 2005;18:273–281. doi:10.1016/j.molcel.2005.04.002. 15866170

[6] Pring M, Evangelista M, Boone C, Yang C, Zigmond SH Mechanism of formin-induced nucleation of actin filaments. Biochemistry. 2003;42:486–496. doi:10.1021/bi026520j. 12525176

[7] Kovar DR, Pollard TD Insertional assembly of actin filament barbed ends in association with formins produces piconewton forces. Proc Natl Acad Sci U S A. 2004;101:14725–14730. doi:10.1073/pnas.0405902101. 15377785 PMC522035

[8] Pruyne D, Evangelista M, Yang C, Bi E, Zigmond S, Bretscher A, Boone C Role of formins in actin assembly: nucleation and barbed-end association. Science. 2002;297:612–615. doi:10.1126/science.1072309. 12052901

[9] Zigmond SH, Evangelista M, Boone C, Yang C, Dar AC, Sicheri F, Forkey J, Pring M Formin leaky cap allows elongation in the presence of tight capping proteins. Curr Biol. 2003;13:1820–1823. doi:10.1016/j.cub.2003.09.057. 14561409

[10] Romero S, Le Clainche C, Didry D, Egile C, Pantaloni D, Carlier MF Formin is a processive motor that requires profilin to accelerate actin assembly and associated ATP hydrolysis. Cell. 2004;119:419–429. doi:10.1016/j.cell.2004.09.039. 15507212

[11] Schönichen A, Geyer M Fifteen formins for an actin filament: a molecular view on the regulation of human formins. ‎Biochim. Biophys. Acta (BBA) Mol Cell Res. 2010;1803:152–163. doi:10.1016/j.bbamcr.2010.01.014. 20102729

[12] Chesarone MA, DuPage AG, Goode BL Unleashing formins to remodel the actin and microtubule cytoskeletons. Nat Rev Mol Biol. 2009;11:62. doi:10.1038/nrm2816. 19997130

[13] Watanabe N, Madaule P, Reid T, Ishizaki T, Watanabe G, Kakizuka A, Saito Y, Nakao K, Jockusch BM, Narumiya S p140mDia, a mammalian homolog of Drosophila diaphanous, is a target protein for Rho small GTPase and is a ligand for profilin. EMBO J. 1997;16:3044–3056. doi:10.1093/emboj/16.11.3044. 9214622 PMC1169923

[14] Liu W, Sato A, Khadka D, Bharti R, Diaz H, Runnels LW, Habas R Mechanism of activation of the Formin protein Daam1. Proc Natl Acad Sci. 2008;105:210. doi:10.1073/pnas.0707277105. 18162551 PMC2224188

[15] Takeya R, Taniguchi K, Narumiya S, Sumimoto H The mammalian formin FHOD1 is activated through phosphorylation by ROCK and mediates thrombin-induced stress fibre formation in endothelial cells. EMBO J. 2008;27:618–628. doi:10.1038/emboj.2008.7. 18239683 PMC2262041

[16] Wang Y, El-Zaru MR, Surks HK, Mendelsohn ME Formin homology domain protein (FHOD1) is a cyclic GMP-dependent protein kinase I-binding protein and substrate in vascular smooth muscle cells. J Biol Chem. 2004;279:24420–24426. doi:10.1074/jbc.M313823200. 15051728

[17] Patel AA, Oztug Durer ZA, van Loon AP, Bremer KV, Quinlan ME Drosophila and human FHOD family formins nucleate actin filaments. J Biol Chem. 2017. 10.1074/jbc.M117.800888 PMC5767859 29127202

[18] Schonichen A, Mannherz HG, Behrmann E, Mazur AJ, Kuhn S, Silvan U, Schoenenberger CA, Fackler OT, Raunser S, Dehmelt L, et al FHOD1 is a combined actin filament capping and bundling factor that selectively associates with actin arcs and stress fibers. J Cell Sci. 2013;126:1891–1901. doi:10.1242/jcs.126706. 23444374

[19] Chhabra ES, Ramabhadran V, Gerber SA, Higgs HN INF2 is an endoplasmic reticulum-associated formin protein. J Cell Sci. 2009;122:1430. doi:10.1242/jcs.040691. 19366733 PMC2721004

[20] Chhabra ES, Higgs HN INF2 is a WASP homology 2 motif-containing formin that severs actin filaments and accelerates both polymerization and depolymerization. J Biol Chem. 2006;281:26754–26767. doi:10.1074/jbc.M604666200. 16818491

[21] Palazzo AF, Cook TA, Alberts AS, Gundersen GG mDia mediates Rho-regulated formation and orientation of stable microtubules. Nat Cell Biol. 2001;3:723–729. doi:10.1038/35087035. 11483957

[22] Wen Y, Eng CH, Schmoranzer J, Cabrera-Poch N, Morris EJS, Chen M, Wallar BJ, Alberts AS, Gundersen GG EB1 and APC bind to mDia to stabilize microtubules downstream of Rho and promote cell migration. Nat Cell Biol. 2004;6:820. doi:10.1038/ncb1160. 15311282

[23] Henty-Ridilla JL, Rankova A, Eskin JA, Kenny K, Goode BL Accelerated actin filament polymerization from microtubule plus ends. Science. 2016;352:1004–1009. doi:10.1126/science.aaf1709. 27199431 PMC5179141

[24] Gaillard J, Ramabhadran V, Neumanne E, Gurel P, Blanchoin L, Vantard M, Higgs HN Differential interactions of the formins INF2, mDia1, and mDia2 with microtubules. Mol Biol Cell. 2011;22:4575–4587. doi:10.1091/mbc.e11-07-0616. 21998204 PMC3226476

[25] Szikora S, Földi I, Tóth K, Migh E, Vig A, Bugyi B, Maléth J, Hegyi P, Kaltenecker P, Sanchez-Soriano N, et al The formin DAAM is required for coordination of the actin and microtubule cytoskeleton in axonal growth cones. J Cell Sci. 2017. doi:10.1242/jcs.203455. 28606990

[26] Gasteier JE, Madrid R, Krautkrämer E, Schröder S, Muranyi W, Benichou S, Fackler OT Activation of the rac-binding partner FHOD1 induces actin stress fibers via a ROCK-dependent mechanism. J Biol Chem. 2003;278:38902–38912. doi:10.1074/jbc.M306229200. 12857739

[27] Ishizaki T, Morishima Y, Okamoto M, Furuyashiki T, Kato T, Narumiya S Coordination of microtubules and the actin cytoskeleton by the Rho effector mDia1. Nat Cell Biol. 2001;3:8–14. doi:10.1038/35050598. 11146620

[28] Gundersen GG, Kalnoski MH, Bulinski JC Distinct populations of microtubules: tyrosinated and nontyrosinated alpha tubulin are distributed differently in vivo. Cell. 1984;38:779–789. doi:10.1016/0092-8674(84)90273-3. 6386177

[29] Westermann S, Weber K Post-translational modifications regulate microtubule function. Nat Rev Mol Biol. 2003;4:938. doi:10.1038/nrm1260. 14685172

[30] Bartolini F, Moseley JB, Schmoranzer J, Cassimeris L, Goode BL, Gundersen GG The formin mDia2 stabilizes microtubules independently of its actin nucleation activity. J Cell Biol. 2008;181:523. doi:10.1083/jcb.200709029. 18458159 PMC2364705

[31] Thurston SF, Kulacz WA, Shaikh S, Lee JM, Copeland JW The ability to induce microtubule acetylation is a general feature of formin proteins. Plos ONE. 2012;7:e48041. doi:10.1371/journal.pone.0048041. 23110170 PMC3480493

[32] Bartolini F, Andres-Delgado L, Qu X, Nik S, Ramalingam N, Kremer L, Alonso MA, Gundersen GG An mDia1-INF2 formin activation cascade facilitated by IQGAP1 regulates stable microtubules in migrating cells. Mol Biol Cell. 2016;27:1797–1808. doi:10.1091/mbc.e15-07-0489. 27030671 PMC4884070

[33] Thomas SG, Calaminus SDJ, Machesky LM, Alberts AS, Watson SP G-protein coupled and ITAM receptor regulation of the formin FHOD1 through Rho Kinase in platelets. J Thromb Haemostasis. 2011;9:1648–1651. doi:10.1111/jth.2011.9.issue-8. 21605332

[34] Burkhart JM, Vaudel M, Gambaryan S, Radau S, Walter U, Martens L, Geiger J, Sickmann A, Zahedi RP The first comprehensive and quantitative analysis of human platelet protein composition allows the comparative analysis of structural and functional pathways. Blood. 2012;120:e73–82. doi:10.1182/blood-2012-04-416594. 22869793

[35] Chen L, Kostadima M, Martens JHA, Canu G, Garcia SP, Turro E, Downes K, Macaulay IC, Bielczyk-Maczynska E, Coe S, et al Transcriptional diversity during lineage commitment of human blood progenitors. Science. 2014;345:1251033. doi:10.1126/science.1251033. 25258084 PMC4254742

[36] de Graaf CA, Choi J, Baldwin TM, Bolden JE, Fairfax KA, Robinson AJ, Biben C, Morgan C, Ramsay K, Ng AP, et al Haemopedia: an expression atlas of murine hematopoietic cells. Stem Rep. 2016;7:571–582. doi:10.1016/j.stemcr.2016.07.007. PMC5031953 27499199

[37] Rowley JW, Oler AJ, Tolley ND, Hunter BN, Low EN, Nix DA, Yost CC, Zimmerman GA, Weyrich AS Genome-wide RNA-seq analysis of human and mouse platelet transcriptomes. Blood. 2011;118:e101–11. doi:10.1182/blood-2011-03-339705. 21596849 PMC3193274

[38] Zeiler M, Moser M, Mann M Copy number analysis of the murine platelet proteome spanning the complete abundance range. Mol Cell Proteomics. 2014;13:3435–3445. doi:10.1074/mcp.M114.038513. 25205226 PMC4256495

[39] Higashi T, Ikeda T, Shirakawa R, Kondo H, Kawato M, Horiguchi M, Okuda T, Okawa K, Fukai S, Nureki O, et al Biochemical characterization of the Rho GTPase-regulated actin assembly by diaphanous-related formins, mDia1 and Daam1, in platelets. J Biol Chem. 2008;283:8746–8755. doi:10.1074/jbc.M707839200. 18218625

[40] Gao G, Chen L, Dong B, Gu H, Dong H, Pan Y, Gao Y, Chen X RhoA effector mDia1 is required for PI 3-kinase-dependent actin remodeling and spreading by thrombin in platelets. Biochem Biophys Res Commun. 2009;385:439–444. doi:10.1016/j.bbrc.2009.05.090. 19470376

[41] Steele BM, Harper MT, Smolenski AP, Alkazemi N, Poole AW, Fitzgerald DJ, Maguire PB WNT-3a modulates platelet function by regulating small GTPase activity. FEBS Lett. 2012;586:2267–2272. doi:10.1016/j.febslet.2012.05.060. 22705156

[42] Peng J, Kitchen SM, West RA, Sigler R, Eisenmann KM, Alberts AS Myeloproliferative defects following targeting of the Drf1 gene encoding the mammalian diaphanous–related formin mDia1. Cancer Res. 2007;67:7565. doi:10.1158/0008-5472.CAN-07-1467. 17699759

[43] Sakata D, Taniguchi H, Yasuda S, Adachi-Morishima A, Hamazaki Y, Nakayama R, Miki T, Minato N, Narumiya S Impaired T lymphocyte trafficking in mice deficient in an actin-nucleating protein, mDia1. J Exp Med. 2007;204:2031. doi:10.1084/jem.20062647. 17682067 PMC2118705

[44] Eisenmann KM, West RA, Hildebrand D, Kitchen SM, Peng J, Sigler R, Zhang J, Siminovitch KA, Alberts AS T cell responses in mammalian diaphanous-related formin mDia1 knock-out mice. J Biol Chem. 2007;282:25152–25158. doi:10.1074/jbc.M703243200. 17595162

[45] Keerthivasan G, Mei Y, Zhao B, Zhang L, Harris CE, Gao J, Basiorka AA, Schipma MJ, McElherne J, Yang J, et al Aberrant overexpression of CD14 on granulocytes sensitizes the innate immune response in mDia1 heterozygous del(5q) MDS. Blood. 2014;124:780–790. doi:10.1182/blood-2014-01-552463. 24891322 PMC4118486

[46] Mei Y, Feng G, Rahimi N, Zhao B, Zhang J, Cao L, Yang J, Gao J, Chen Y, Sumagin R, et al Loss of mDia1 causes neutropenia via attenuated CD11b endocytosis and increased neutrophil adhesion to the endothelium. Blood Adv. 2017;1:1650–1656. 29296812 10.1182/bloodadvances.2017007906 PMC5728338

[47] Shi Y, Zhang J, Mullin M, Dong B, Alberts AS, Siminovitch KA The mDial formin is required for neutrophil polarization, migration, and activation of the LARG/RhoA/ROCK signaling axis during chemotaxis. J Immunol. 2009;182:3837. doi:10.4049/jimmunol.0803838. 19265163

[48] Pan J, Lordier L, Meyran D, Rameau P, Lecluse Y, Kitchen-Goosen S, Badirou I, Mokrani H, Narumiya S, Alberts AS, et al The formin DIAPH1 (mDia1) regulates megakaryocyte proplatelet formation by remodeling the actin and microtubule cytoskeletons. Blood. 2014;124:3967. doi:10.1182/blood-2013-12-544924. 25298036

[49] Bender M, Thon JN, Ehrlicher AJ, Wu S, Mazutis L, Deschmann E, Sola-Visner M, Italiano JE, Hartwig JH Microtubule sliding drives proplatelet elongation and is dependent on cytoplasmic dynein. Blood. 2015;125:860–868. doi:10.1182/blood-2014-09-600858. 25411426 PMC4311231

[50] Stritt S, Nurden P, Turro E, Greene D, Jansen SB, Westbury SK, Petersen R, Astle WJ, Marlin S, Bariana TK, et al A gain-of-function variant in DIAPH1 causes dominant macrothrombocytopenia and hearing loss. Blood. 2016;127:2903. doi:10.1182/blood-2015-10-675629. 26912466

[51] Neuhaus C, Lang-Roth R, Zimmermann U, Heller R, Eisenberger T, Weikert M, Markus S, Knipper M, Bolz HJ Extension of the clinical and molecular phenotype of DIAPH1-associated autosomal dominant hearing loss (DFNA1). Clin Genet. 2017;91:892–901. doi:10.1111/cge.12915. 27808407

[52] Ajima R, Bisson JA, Helt J-C, Nakaya M-A, Habas R, Tessarollo L, He X, Morrisey EE, Yamaguchi TP, Cohen ED DAAM1 and DAAM2 are co-required for myocardial maturation and sarcomere assembly. Dev Biol. 2015;408:126–139. doi:10.1016/j.ydbio.2015.10.003. 26526197 PMC4765503

[53] Kan-O M, Takeya R, Abe T, Kitajima N, Nishida M, Tominaga R, Kurose H, Sumimoto H Mammalian formin Fhod3 plays an essential role in cardiogenesis by organizing myofibrillogenesis. Biology Open. 2012;1:889. doi:10.1242/bio.20121370. 23213483 PMC3507241

[54] Li D, Hallett MA, Zhu W, Rubart M, Liu Y, Yang Z, Chen H, Haneline LS, Chan RJ, Schwartz RJ, et al Dishevelled-associated activator of morphogenesis 1 (Daam1) is required for heart morphogenesis. Development. 2011;138:303–315. doi:10.1242/dev.055566. 21177343 PMC3005605

[55] Ushijima T, Fujimoto N, Matsuyama S, Kan-O M, Kiyonari H, Shioi G, Kage Y, Yamasaki S, Takeya R, Sumimoto H The actin-organizing formin protein Fhod3 is required for postnatal development and functional maintenance of the adult heart in mice. J Biol Chem. in press;293:148–162. doi:10.1074/jbc.M117.813931. 29158260 PMC5766910

[56] Dumont J, Million K, Sunderland K, Rassinier P, Lim H, Leader B, Verlhac M-H Formin-2 is required for spindle migration and for the late steps of cytokinesis in mouse oocytes. Dev Biol. 2007;301:254–265. doi:10.1016/j.ydbio.2006.08.044. 16989804

[57] Leader B, Lim H, Carabatsos MJ, Harrington A, Ecsedy J, Pellman D, Maas R, Leder P Formin-2, polyploidy, hypofertility and positioning of the meiotic spindle in mouse oocytes. Nat Cell Biol. 2002;4:921. doi:10.1038/ncb880. 12447394

[58] Ji P, Jayapal SR, Lodish HF Enucleation of cultured mouse fetal erythroblasts requires Rac GTPases and mDia2. Nat Cell Biol. 2008;10:314. doi:10.1038/ncb1693. 18264091

[59] Li X, Mei Y, Yan B, Vitriol E, Huang S, Ji P, Qiu Y Histone deacetylase 6 regulates cytokinesis and erythrocyte enucleation through deacetylation of formin protein mDia2. Haematologica. 2017;102:984–994. doi:10.3324/haematol.2016.161513. 28255013 PMC5451330

[60] Mei Y, Zhao B, Yang J, Gao J, Wickrema A, Wang D, Chen Y, Ji P Ineffective erythropoiesis caused by binucleated late-stage erythroblasts in mDia2 hematopoietic specific knockout mice. Haematologica. 2016;101:e1–e5. doi:10.3324/haematol.2015.134221. 26471482 PMC4697897

[61] Brown EJ, Schlöndorff JS, Becker DJ, Tsukaguchi H, Uscinski AL, Higgs HN, Henderson JM, Pollak MR Mutations in the formin protein INF2 cause focal segmental glomerulosclerosis. Nat Genet. 2010;42:72–76. doi:10.1038/ng.505. 20023659 PMC2980844

[62] Subramanian B, Sun H, Yan P, Charoonratana VT, Higgs HN, Wang F, Lai K-MV, Valenzuela DM, Brown EJ, Schlöndorff JS, et al Mice with mutant Inf2 show impaired podocyte and slit diaphragm integrity in response to protamine induced kidney injury. Kidney Int. 2016;90:363–372. doi:10.1016/j.kint.2016.04.020. 27350175 PMC5363079

[63] Rossi A, Kontarakis Z, Gerri C, Nolte H, Hölper S, Krüger M, Stainier DYR Genetic compensation induced by deleterious mutations but not gene knockdowns. Nature. 2015;524:230. doi:10.1038/nature14580. 26168398

[64] Isogai T, van der Kammen R, Leyton-Puig D, Kedziora KM, Jalink K, Innocenti M Initiation of lamellipodia and ruffles involves cooperation between mDia1 and the Arp2/3 complex. J Cell Sci. 2015. doi:10.1242/jcs.176768. 26349808

[65] Bender M, Stritt S, Nurden P, van Eeuwijk JMM, Zieger B, Kentouche K, Schulze H, Morbach H, Stegner D, Heinze KG,et al Megakaryocyte-specific Profilin1-deficiency alters microtubule stability and causes a Wiskott–aldrich syndrome-like platelet defect. Nat Commun. 2014;5:4746. doi:10.1038/ncomms5746. 25187265

[66] Aguilar A, Pertuy F, Eckly A, Strassel C, Collin D, Gachet C, Lanza F, Leon C Importance of environmental stiffness for megakaryocyte differentiation and proplatelet formation. Blood. 2016;128:2022–2032. doi:10.1182/blood-2016-02-699959. 27503502

[67] Lee C-y, Lou J, Wen K-K, McKane M, Eskin SG, Rubenstein PA, Chien S, Ono S, Zhu C, McIntire LV Regulation of actin catch-slip bonds with a RhoA-formin module. Sci Rep. 2016;6:35058. doi:10.1038/srep35058. 27731359 PMC5059732

[68] Campellone KG, Welch MD A nucleator arms race: cellular control of actin assembly. Nat Rev Mol Cell Biol. 2010;11:237–251. doi:10.1038/nrm2867. 20237478 PMC2929822

[69] DeWard AD, Alberts AS Microtubule stabilization: formins assert their independence. Curr Biol. 2008;18:R605–8. doi:10.1016/j.cub.2008.06.001. 18644336

[70] Foldi I, Szikora S, Mihaly J Formin’ bridges between microtubules and actin filaments in axonal growth cones. Neural Regen Res. 2017;12:1971–1973. doi:10.4103/1673-5374.221148. 29323030 PMC5784339

